# A Model for Genetic and Epigenetic Regulatory Networks Identifies Rare Pathways for Transcription Factor Induced Pluripotency

**DOI:** 10.1371/journal.pcbi.1000785

**Published:** 2010-05-13

**Authors:** Maxim N. Artyomov, Alexander Meissner, Arup K. Chakraborty

**Affiliations:** 1 Department of Chemistry, Massachusetts Institute of Technology, Cambridge, Massachusetts, United States of America; 2 Broad Institute of MIT and Harvard, Cambridge, Massachusetts, United States of America; 3 Department of Stem Cell and Regenerative Biology, Harvard University and Harvard Stem Cell Institute, Cambridge, Massachusetts, United States of America; 4 Department of Chemical Engineering, Massachusetts Institute of Technology, Cambridge, Massachusetts, United States of America; 5 Department of Biological Engineering, Massachusetts Institute of Technology, Cambridge, Massachusetts, United States of America; 6 Ragon Institute of MGH, MIT, and Harvard, Charlestown, Massachusetts, United States of America; Johns Hopkins University, United States of America

## Abstract

With relatively low efficiency, differentiated cells can be reprogrammed to a pluripotent state by ectopic expression of a few transcription factors. An understanding of the mechanisms that underlie data emerging from such experiments can help design optimal strategies for creating pluripotent cells for patient-specific regenerative medicine. We have developed a computational model for the architecture of the epigenetic and genetic regulatory networks which describes transformations resulting from expression of reprogramming factors. Importantly, our studies identify the rare temporal pathways that result in induced pluripotent cells. Further experimental tests of predictions emerging from our model should lead to fundamental advances in our understanding of how cellular identity is maintained and transformed.

## Author Summary

Most cells in an organism have the same DNA. Yet, different cell types express different proteins and carry out different functions. These differences are reflected by cell epigenetics; i.e., DNA in different cell types is packaged distinctly, making it hard to express certain genes while facilitating the expression of others. During development, upon receipt of appropriate cues, pluripotent embryonic stem cells differentiate into diverse cell types that make up the organism (e.g., a human). There has long been an effort to make this process go backward— i.e., reprogram a differentiated cell (e.g., a skin cell) to pluripotent status. Recently, this has been achieved by overexpressing specific transcription factors in differentiated cells. This method does not use embryonic material and promises the development of patient-specific regenerative medicine. The mechanisms that make reprogramming rare, or even possible, are poorly understood. We have developed the first computational model of transcription factor-induced reprogramming. Results obtained from the model are consistent with diverse observations, and identify the rare pathways that allow reprogramming to occur. If validated by further experiments, our model could be further developed to design optimal strategies for reprogramming and shed light on basic questions in biology.

## Introduction

Cellular states are plastic, and even terminally differentiated cells (e.g., B-cells) can be reprogrammed to pluripotency by ectopic expression of selected transcription factors [1], [2], [3], [4], [5], [6], [7]. This finding raises the possibility of creating patient-specific stem cells for regenerative medicine [8]. However, reprogramming efficiencies range from 0.0001% to 29% [5], [6], [9], [10], with most reports showing that successful induction of the pluripotent state is rare even if all required factors are present [11], [12]. The genetic and epigenetic regulatory mechanisms that make reprogramming possible, and determine its efficiency, are poorly understood [2]. Elucidating these mechanistic principles can help define optimal strategies for reprogramming differentiated cells, and answer fundamental questions regarding how cellular identity is maintained and transformed.

In spite of recent progress, our knowledge of the identities and functions of the genes and proteins involved in regulating the transformation of cellular identity is grossly incomplete [2], [13], [14]. Thus, it is not yet possible to construct a detailed molecular mechanistic description of how epigenetic modifications and expression of master regulatory genes are controlled. However, ectopic expression of the same transcription factors can reprogram different cell types [1], [6], [12], and the genetic and epigenetic transformations observed during reprogramming of diverse differentiated cells share many common features [2], [11], [15], [16], [17], [18], [19]. These common observations can be the basis for developing a conceptual understanding of the general architecture of the genetic and epigenetic networks that regulate transcription factor induced reprogramming and establish cellular identity during differentiation.

We have taken a step toward this goal by developing a computational model that is consistent with, and suggests general mechanistic explanations for, empirical observations of transcription factor induced reprogramming. The model makes experimentally-testable predictions. If validated, descendents of this model could also provide insights into the aberrant de-differentiation events which characterize some of the most malignant cancers.

## Results

### Model development

Elegant theoretical models for the molecular regulatory networks responsible for stem cell renewal and differentiation and the population dynamics of these processes have been created [20], [21], [22], [23], [24]. Our goal is different. We aim to develop a model for the architecture of coupled epigenetic and genetic networks which describes large changes in cellular identity (e.g., induction of pluripotency by reprogramming factors). Although the general principles of interactions between genetic and epigenetic layers of regulation have been described [25], [26], no computational model has been developed to study the outcomes of such interactions and their biological consequences. Such a computational model would be a useful complement to experiments in understanding the processes that occur during reprogramming of differentiated cells, and why reprogramming is rare. Here, we propose, to our knowledge, the first computational model that describes how cellular identity changes by creating a mathematical description of interactions between epigenetic and genetic networks. Our goal is not to describe the details of how specific regulatory proteins interact, but rather, to understand general principles underlying how cellular states evolve upon ectopic expression of certain types of genes. The concise model we have developed explains why reprogramming probability is low, and makes experimentally testable predictions.

**Figure 1:**
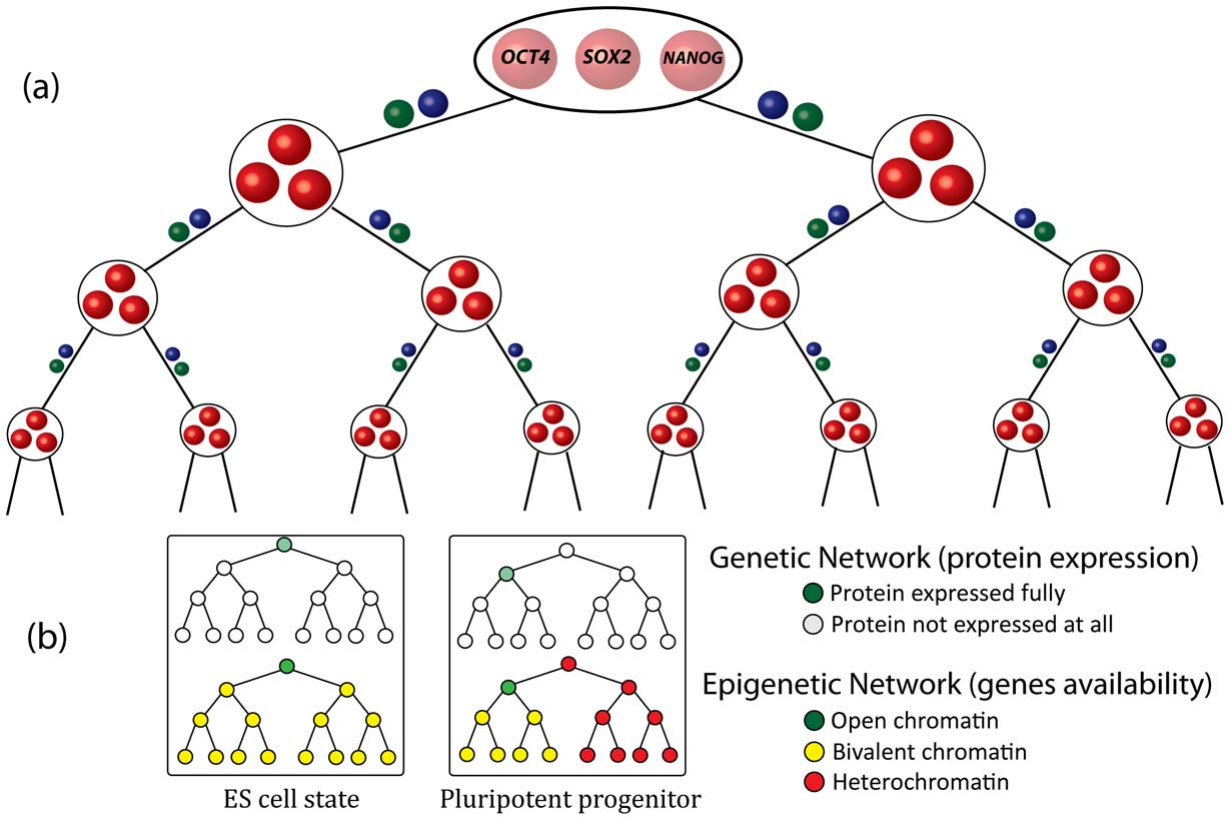
Specification of the genetic and epigenetic states that describe cellular states. (a) Only the master-regulatory genes that govern cell state are arranged in a hierarchy (house keeping, stress-response and many other genes are not considered). Each node of the hierarchy represents an ensemble of master-regulatory genes that govern a particular cellular state. For example, genes in the top node are known master-regulators of the embryonic stem cell state (e.g. Oct4, Sox2, Nanog). When a cell is in the ES state, only these three genes will be expressed while other genes will not. Similarly, when a cell is fully differentiated, genes in one of the bottom modules will be expressed but not any other gene in the network. Each master-regulatory ensemble can contain many genes, only three are shown in each node. Green and blue balls above the links indicate that not only master regulatory proteins but also other proteins such as chromatin modifiers and housekeeping genes mediate interactions between modules of master-regulators. (b) Fig. 1a has been coarse-grained such that only master-regulatory modules (nodes in fig. 1a) are shown. Cellular identity is determined by both epigenetic (chromatin marks, DNA methylation) and genetic (expression profile) states. Examples of two states (ES state and “left” pluripotent progenitor) are shown. For each example, two lattices are needed to describe the state of gene expression and the epigenome: top lattice reflects the expression levels of master-regulatory proteins in the ES/progenitor state and bottom lattice reflects the epigenetic state of master-regulatory genes in the ES/progenitor state.

Almost all cells in a multi-cellular organism share the same DNA sequence. Yet, different cell types express distinct genes and perform different functions. Epigenetic modifications are major regulators of cell-type specific gene expression. They function by packaging DNA into configurations that allow only some genes to be expressed, while other genes are tightly packed into heterochromatin structures that hinder access of most transcription factors [27]. Changes in cellular identity during developmental differentiation or transcription factor induced reprogramming require modification of the epigenetic state of the cell. The maintenance and alteration of cellular identity is regulated by a complex set of interactions between developmentally important genes, chromatin modifiers, transcription factors *etc.*, the details of which remain unknown. Toward developing a model for the architecture of these complex regulatory networks we consider only the developmentally important genes. For simplicity, each ensemble of genes responsible for maintenance of a particular cellular identity (e.g., Oct4, Sox2, etc., for pluripotency) is described as a single module (Fig. 1a). Theoretical justification for treating genes that control the embryonic stem (ES) cell state as a collective unit exists [28]. We also carried out some studies with each module consisting of a small number of genes (see corresponding discussion below).

ES cells can differentiate into various lineages. Upon further differentiation, cells become more restricted. For example, hematopoetic stem cells can differentiate into T and B-lymphocytes, but not neural cells. Therefore, in our model, we arrange gene modules in a hierarchy (Fig. 1a). Although each cell state can potentially differentiate into many branches, without loss of generality, we consider two branches to emanate from each cell state. Thus, the cellular states are arranged on a Cayley tree. In our model, a cell state (Fig. 1b) is specified by: i] the state of the epigenome, and ii] the expression levels of master regulatory genes.

#### Specification and regulation of the epigenome

The epigenome is specified by chromatin states. Histones with positive marks (e.g. H3K4me3) promote transcription, and histones with negative marks (e.g. H3K27me3) repress transcription [29], [30]. Hypermethylated genes are also silent [17], [31]. Genes associated with both H3K4me3 and H3K27me3 simultaneously (bivalent marks) can recruit promoters, but transcription is suppressed [32], [33], [34]. Based on these observations, in our model, each developmentally important gene module can adopt one of three possible epigenetic states. It can be silent either due to negative histone marks or DNA methylation (denoted as the “−1” state), marked positively by histone marks (denoted as the “+1” state), and marked bivalently (denoted as the “0” state). From the standpoint of gene expression, each module can be either actively transcribing (denoted as the “+1” state) or not (denoted as the “0” state).

**Figure 2:**
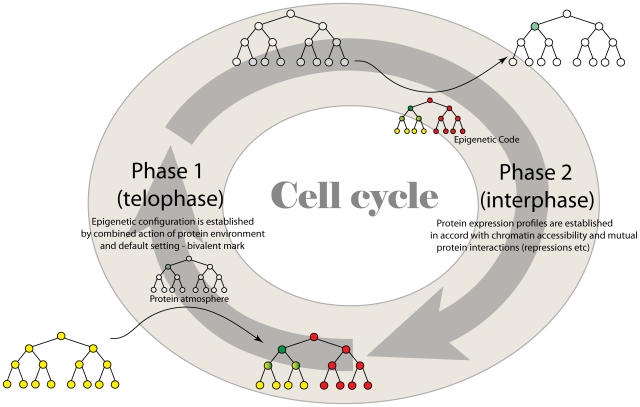
Simplified model for progression through the cell cycle. The cell cycle is divided into two generalized phases: called interphase and telophase for simplicity. Gene expression occurs during the interphase, while cell division and associated processes occur in the telophase. In the interphase gene expression profile is governed by the stable epigenetic marks on the master-regulatory genes. In the telophase, however, protein environment can change the epigenetic marks of the master-regulaory genes, particularly when DNA is decondensing after cell division. Differentiation signals (newly expressed proteins) determine future epigenetic marks created during telophase due to the action of the new protein environment. The color code representing genetic and epigenetic states is the same as in Fig. 1.

During interphase, DNA with genes packaged in a way characteristic of the cell's identity manages gene transcription and protein synthesis. Before cell division, the chromosomes condense. During telophase at the end of mitosis, the prevailing protein environment could alter the chromatin states of decondensing chromosomes in a daughter cell, thereby modifying the epigenetic state of its DNA [15], [35]. We divide the cell cycle into two parts (Fig. 2). During phase one (termed interphase, for ease of reference), the epigenetic state cannot be modified and gene expression is subject to this constraint. In phase two (termed telophase, for ease of reference), the epigenetic state can potentially be altered by the protein environment established during the preceding interphase.

Chromatin state maps show that the ES state is characterized by an unusually large proportion of bivalent chromatin marks on developmentally important genes [19], [32], [36]. Therefore, we define the ES state as one where the gene module controlling this state (expressing Sox2, Oct4, etc.) is in the open chromatin state and all other master regulator genes are bivalently marked (Fig. 1b, left panel). Since the identities of all master-regulatory genes are not yet experimentally available, it should be noted that bivalency of *all* master-regulatory modules in the ES state is an assumption that extrapolates available knowledge to yet unidentified modules.

**Figure 3:**
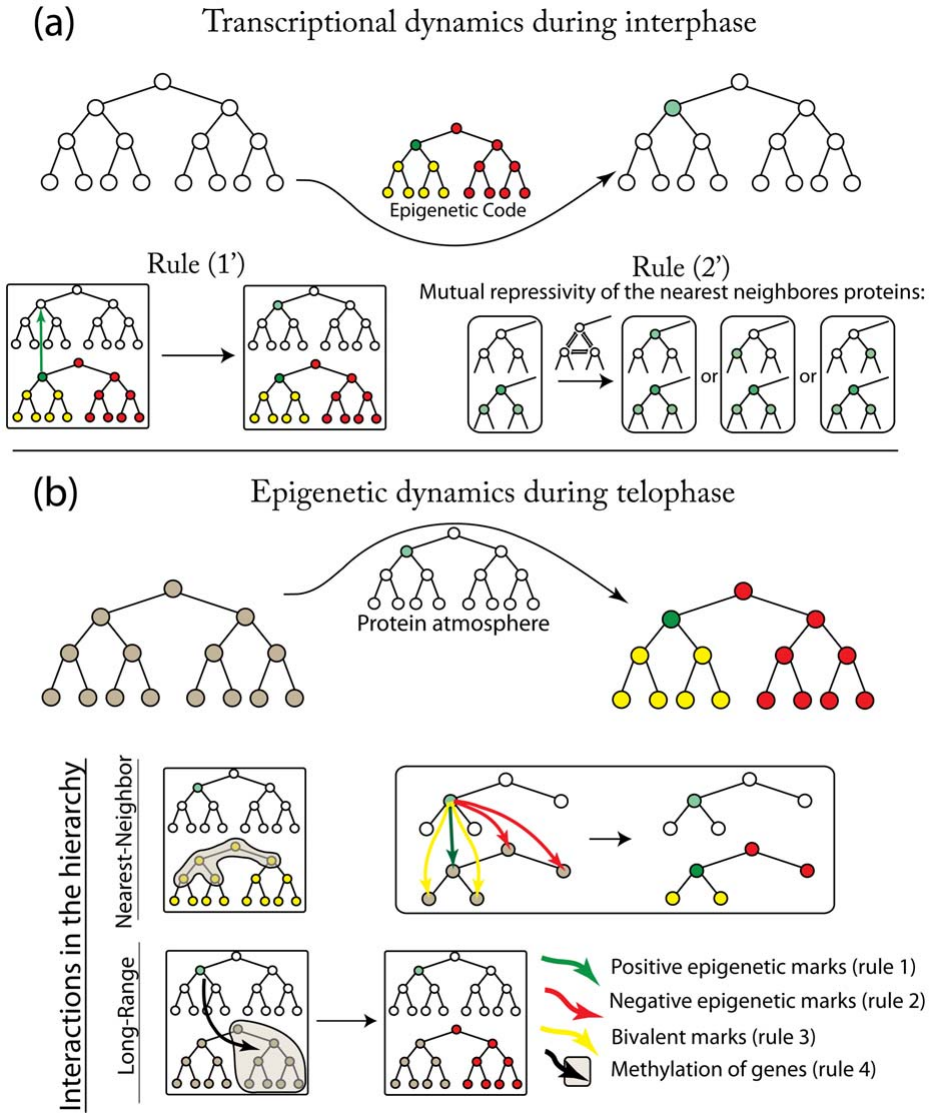
Rules that govern interactions within epigenetic and genetic networks. (a) During interphase, gene expression profiles of master-regulatory modules are established. Gene expression is influenced by epigenetic marking of the corresponding gene and interactions between expressed proteins. Two rules reflect this in our simulation: 1) when master-regulatory gene is in epigenetically marked positively, it favors expression of the corresponding protein; 2) when two (three) neighboring genes are in epigenetically open states, they all favor expression of corresponding proteins, but due to their mutually repressive action (see text) only one of two(three) genes are expressed. Which gene is expressed is chosen stochastically. (b) During the telophase, the protein environment can alter the epigenetic marks on the master-regulatory genes. Epigenetic marks on both neighboring and distant genes in the hierarchy can be altered. Long-range effect is typically mediated through DNA methylation which epigenetically silences all of the master-regulatory genes of unrelated lineages and also ancestral states (see text). Short-range interactions affect nearest-neighbors differentially: progenies master-regulatory genes are preferentially put into bivalent states while progenitor and competing lineage modules are epigenetically silenced. The color code representing genetic and epigenetic states is the same as in Fig. 1. The numbers corresponding to the rules are the same as in text and Table 1.

It is known that, as cells differentiate from the ES state, bivalently marked genes remain bivalent, acquire a positive mark, or are silenced by negatively marked histones or methylation [19], [32], [36]. Other than pluripotent ES cells, upon receiving appropriate cues, a cell state can only differentiate into other states in the same lineage. Upon differentiation from the ES state positive histone marks are removed at an earlier stage compared to silencing of genes by DNA methylation, and reactivation of DNA methylated genes is more difficult than those with negative histone marks (summarized in Table 1). These facts are encapsulated in our model by the following rules regarding how proteins expressed by a particular gene module can modify epigenetic states during telophase (Fig. 3b): 1] They favor putting positive marks on the module that expresses them, which enables stable maintenance of cellular identity. 2] They favor putting negative histone marks on the modules regulating the immediate progenitor or an immediate “sibling” in the hierarchy; this hinders differentiation into cells in competing lineages and accidental de-differentiation to the progenitor. 3] They favor putting bivalent histone marks on the modules that regulate immediate progeny, which keeps cells poised to differentiate. 4] They favor methylation of all modules that regulate cell states in competing lineages or less differentiated states in the same lineage. This has a similar effect as the marking of histones in rule 2.

**Table 1:** Summary of the rules governing interactions between genetic and epigenetic networks during the two phases labeled interphase and telophase (details in text).

During Interphase:	During Telophase: proteins expressed by a gene module…
(1′) if a gene module is positively marked, its expression is favored. Expression of bivalently marked gene modules is not favored, but it is not as strongly suppressed as modules that are negatively marked or DNA methylated	(1) … favor putting positive marks on the module that expresses them, which enables stable maintenance of cellular identity (2) … favor putting negative histone marks on the modules regulating the immediate progenitor or an immediate “sibling” in the hierarchy
(2′) Diverse experimental data [38], [39] show that, due to effects such as feedback regulation, etc., expression of genes from competing lineages is mutually repressed.	(3) … favor putting bivalent histone marks on modules regulating immediate progeny (4) … favor methylation of all modules that regulate cell states in competing lineages or less differentiated states in the same lineage

Rules 1–3 are based on experimental facts, and concern how proteins expressed by a gene module can affect the histone marks of only modules that regulate its immediate precursor, immediate progeny (see Fig. 3b), or other states to which its precursor can differentiate (i.e., “nearest neighbors” on the hierarchy of gene modules shown in Fig. 1a).

Rule 4 states that proteins expressed by a gene module favor silenced chromatin state of gene modules that are distal from it in the hierarchy by DNA methylation (Fig. 3b). Although there are no experimental measurements showing that methylation of unrelated lineages is directly caused by master-regulatory genes of current cell state, this rule is motivated by the global DNA methylation of genes of unrelated lineages observed upon cell differentiation [16], [17] and the fact that global DNA hypomethylation blocks differentiation [37]. To further investigate the effect of such long-range interactions, we have perturbed the formulation of rule 4 in different ways. We find that unless the long-ranged nature of rule 4 is included, the *in silico* reprogramming trajectories exhibit features which are inconsistent with experimental observations. In particular, stable expression of protein products of the ES master-regulatory module becomes possible within the first reprogramming cycle, in contradiction with the observation that endogenous Oct4 is expressed shortly before completion of reprogramming after at least 12 days of action of reprogramming factors (see, for example Fig. 2 in [2] and references therein). Our computational results are also inconsistent with this observation if we allow proteins expressed by a module to put bivalent marks on all modules that regulate states in the lineage that are below it, rather than just the immediate progeny (rule 3 above).

#### Specification and regulation of gene expression

In our model, gene expression during interphase is subject to constraints imposed by the epigenetic marks as follows (summarized in Table 1): 1′] if a gene module is positively marked, its expression is favored. Expression of bivalently marked gene modules is not favored, but it is not as strongly suppressed as modules that are negatively marked or DNA methylated (see Eq. 3 in Methods). 2′] Diverse experimental data [38], [39] show that, due to effects such as feedback regulation, etc., expression of genes from competing lineages is mutually repressed. For example, GATA-1, erythroid lineage specific gene, and PU-1, transcription factor for genes of myeloid lineage are among the most studied master-regulatory genes. They posses typical properties attributed to the master-regulators in this manuscript: they enhance their own expression [40], [41] and mutually antagonize each others' activity [20], [38], [42]. We thus impose such mutually repressive interactions to gene modules that regulate directly competing cellular states (i.e., nearest neighbors in the hierarchy in Fig. 3a).

Rules 1–4 (summarized in Table 1) noted above are meant to describe how the epigenetic state is maintained and how it could evolve due to protein products of signaling events or ectopic expression of transcription factors. During telophase, there could be a “tug of war” between the epigenetic state preferred by newly expressed proteins and that preferred by proteins expressed in accord with the preceding epigenetic state [35]. Similarly, rules 1′ and 2′ could lead to a tug of war between expression of different genes. Our computations reveal possible outcomes of these battles.

The epigenetic modifications during telophase or gene expression patterns during interphase are simulated on a computer using a Monte-Carlo algorithm, with rules 1–4 and 1′–2′ represented as effective Hamiltonians (Eqs. 2–3, Methods). We specify the initial epigenetic state of the cell or the proteins that have been expressed in the previous interphase (including signaling products and ectopic expression of transcription factors). If the gene expression pattern is specified, simulation of telophase results in an epigenetic state that becomes the input for simulation of the next interphase, and so on (see Methods).

### Differentiation

**Figure 4:**
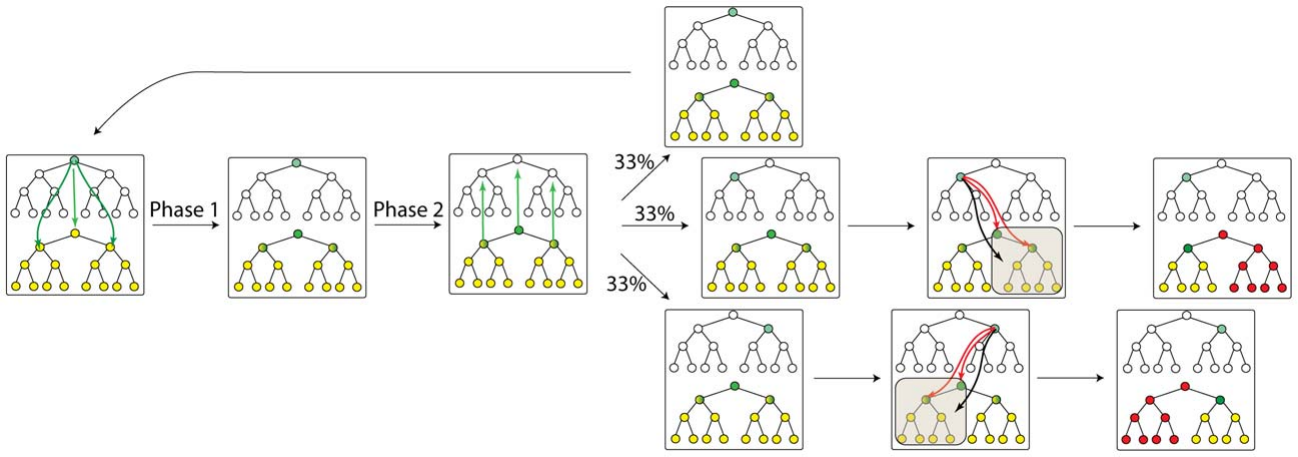
Changing cellular identity during self-initiated differentiation of the ES cell-state. Process begins with cell division where regulatory modules of progenies are put into epigenetically open states. In phase 2 only one of the three neighboring proteins can be actually expressed in accord with Fig. 3a. Thus, one of three possibilities is realized: self-renewal, and differentiation to the “left” or “right” lineages. In the absence of external stimuli, in our simulations, there is an equal chance to observe each outcome. Simulations are performed with parameter values F = 2000; J = 3000; G = 25; H = 40; a = 0; b = 0.3. The color code representing genetic and epigenetic states is the same as in Fig. 1.

ES cells are cultured in specific media (e.g., containing LIF/BMP4 for mouse ES cells) to prevent differentiation [43]. The medium inhibits a self-induced differentiation pathway. We represent this feature by assuming that proteins expressed by the module regulating the ES state favor putting positive chromatin marks on gene modules regulating immediate progenies if LIF, etc. are absent. Simulations of this situation show (Fig. 4) that, as in experiments [2], ES cells differentiate randomly to one of their progeny.

Our model exhibits robust differentiation (forward programming) to specific cell states when the appropriate cues are delivered. Appropriate cues are expression of proteins (e.g., signaling products) that become available during interphase. In the next telophase, these proteins favor putting positive histone marks on the gene module regulating the appropriate progeny of the current cellular state (rule 1). Results from our computer simulations (Fig. 4 in Text S1) demonstrate that our model exhibits high-fidelity responses to such differentiation cues. This is consistent with the experimental observation that overexpression of the master-regulatory genes of desired lineage leads to predominant differentiation in that direction [44], [45]. This result is relevant because practical use of induced pluripotent cells will involve differentiating them to desired cell types. We also find an exponential decay of the number of progenitor cells (with a signal strength-dependent lifetime), as has been noted before [46].

### Reprogramming

We simulate reprogramming experiments by starting with a terminally differentiated cell state where genes from other lineages, etc., have been epigenetically silenced. Our basic premise is that terminally differentiated cells can reprogram because protein products of the ectopically expressed genes can potentially alter the epigenetic state of the cell as a cell progresses through the telophase. In our low resolution model, we identify genes not by names, but rather by their functional properties. We presume that Klf4 and c-Myc are important ingredients of the reprogramming “cocktail” because they promote progression through the cell cycle, and this provides more opportunities for the other reprogramming factors to perturb the epigenome during telophase. This functional identification of Klf4 and c-Myc makes our model general, and is validated by experiments showing that shutting down p53 abrogates the need for Klf4 and c-Myc for reprogramming (only Oct4 and Sox2 required) precisely because this also allows faster progression through the cell cycle [47], [48], [49], [50], [51]. (Interestingly, simulataneous action of c-Myc and p53 knock-down decreases the efficiency of reprogramming indicating existence of the optimum). Oct4 and Sox2 have an enormous number of binding targets on the DNA, and are responsible for maintenance of the ES state which likely implies multiple interactions with master-regulatory genes. We therefore identify the ectopic expression of these genes with the function of being highly likely to perturb the epigenome during telophase.

Each gene module in our model corresponds to an ensemble of carefully tuned mutually interacting master-regulatory genes that govern a particular cellular identity. At the moment, not all of the master-regulatory genes of cellular states are experimentally identified, thus we use gene modules to represent these ensembles in a general way. Even though products of ectopically expressed Oct4 and Sox2 have numerous targets [52], it is unlikely that the epigenetic state of many such sets of genes will be simultaneously altered. Thus, in order to mimic the effect of reprogramming factors, we randomly pick one epigenetically silenced gene module and change its state to correspond to open chromatin. To examine the effects of overexpression of ectopic genes, we also study the consequences of multiple epigenetic transformations at a time (see discussions below).

Starting with a terminally differentiated state we perturb the epigenome as described above, and then simulate the next gene expression phase where both the module regulating the terminally differentiated state and the one which was transformed to open chromatin status can express proteins according to rules 1′–2′ (or Eq. 3). The protein atmosphere thus generated becomes the input to simulation of the next telophase according to rules 1–4 (or Eq. 2). This can then potentially establish a new epigenetic state which becomes input to simulation of the next gene expression phase; i.e., the genetic and epigenetic states are allowed to come to a new balance. Then, the epigenetic state of another randomly picked silent gene module is changed to open chromatin because of the effects of reprogramming factors. This procedure is continued until a fully reprogrammed or a dead/arrested state is achieved (see below).

We carried out 10, 000 independent replicate simulations of the effects of ectopic expression of reprogramming factors on a differentiated cell in a model with four levels in the hierarchy of cellular states. Results from each simulation describe the fate of a single cell in a population. Only 3 out of 10, 000 “cells” successfully reprogrammed; i.e, as in experiments, reprogramming is rare. The percentage of cells that reprogram depends upon the number of levels in the hierarchy (0.0001% and 2% of the cells reprogram successfully for a five-level and three-level hierarchy, respectively). This suggests that reprogramming efficiency should improve for less differentiated cells. This has been demonstrated directly in a well-defined lineage such as the hematopoietic system [53]. Additionally, Hanna et al. demonstrated a notable increase in the efficiency of reprogramming B cells upon Pax5 knockdown [12]. Loss of Pax5 had been previously shown to cause dedifferentiation of B cells to a common progenitor that upon transplantation allowed T cell development [54].

We report results for models consisting of 3-, 4- and 5-levels in the hierarchy of gene modules, but in real organisms the depth of the differentiation tree could be as large as tens of levels [55]. Since our results indicate that reprogramming efficiency decreases quickly with the increase in the depth of the hierarchy, it is natural to ask why reprogramming is at all feasible. The reason is that master-regulatory genes that regulate closely related states are not mutually exclusive sets of genes. The difference between genes that regulate closely related cellular states can be as small as one or two genes [54]. However, genes that regulate cellular states distal in the hierarchy are not correlated in this way. As our model does not treat correlations between genes that regulate closely related states, in effect, each gene module in our model represents master regulatory genes that control the identity of a number of cellular states that have many master regulatory genes in common. Thus, a 5-level hierarchy in our model might represent a 50-level depth of differentiation in a real organism.

The results reported above were obtained for specific values of parameters (Table 2) which represent rules 1–4 and 1′–2′ (Eqs., 2–3 in Methods). Our simulation results are consistent with diverse experimental observations (see Table 3 and discussion below) only if the methylation constraints (rule 4) and mutual repression of expression of gene modules (rule 2′) are relatively strong effects (i.e. H>G and J>F, see Table 2, Eqns (2–3), and parameter sensitivity in SI for further details). As long as these two conditions are met, the specific choice of parameter values only alters the quantitative value of the number of successfully reprogrammed cells, but reprogramming to the ES state remains rare.

**Table 2:** Parameters used to obtain the simulation results reported in the main text.

Parameter of the model	Value of the parameter
Protein action on epigenetic lattice	G = 25
Mutual suppression by two proteins	J = 3000
Action from epigenetic to genetic lattice	F = 2000
Methylation strength	H = 40
Minimal protein expression level required to actively affect epigenetic state of the gene	a = 0
Minimal epigenetic availability of the gene required to allow protein expression	b = 0.3

Results do not change qualitatively as long as the parameters lie in the following ranges: H>G; J>F≫H,G; 0.1<*b*<0.5 and 0<*a*<0.6.

**Table 3:** Examples of experimental features of reprogramming explained by the proposed model (see details in the text).

Experimental observation	Explanation
Reprogramming takes *at least* 12 days of continuous exposure to reprogramming factors [11]	In the simulations, reprogramming does not occur in small number of cell divisions. This is because the most probable paths of reprogramming involve a sequence of de-differentiation events to closely related cellular states (see Fig. 5 for details)
Stochastic nature of reprogramming [56]	In the simulations, trajectories starting from identical differentiated state may or may not undergo successful reprogramming. A trajectory in the simulation corresponds to the processes in a single cell.
Low yield of reprogramming process [2]	In the simulations, the majority of cells end up in dead/arrested state with only a few that are successfully reprogrammed
The fact that the same gene cocktail can reprogram different terminal cell types [1], [12]	In the simulations, and the mechanism we propose, exogenously added genes must have multiple targets (e.g., Oct4, Sox2) enabling random epigenetic perturbation of these targets regardless of the original differentiated state. cMyc and Klf-4 enhance progression through the cell cycle, thereby providing opportunities for random epigenetic perturbations.
An increase in the efficiency of reprogramming B cells upon Pax5 knockdown [12]. (Loss of Pax5 had been previously shown to cause dedifferentiation of B cells)	In the simulations, trajectories starting from the less differentiated state (i.e., higher up in the cell hierarchy) have higher chance to undergo successful reprogramming, thus making reprogramming more efficient compared to reprogramming of the less differentiated state.

**Figure 5:**
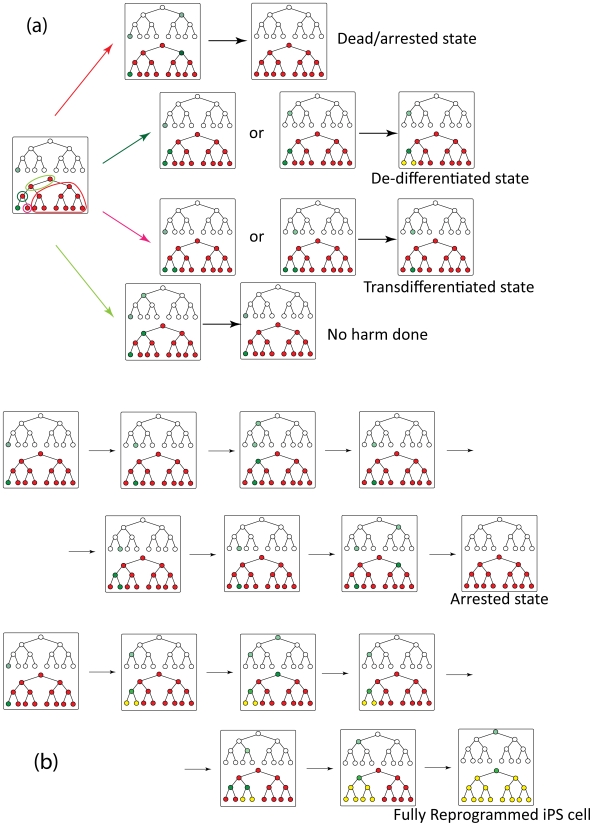
Reprogramming is a consequence of random perturbation of epigenetic state of the cell. In our model, reprogramming factors can change the epigenetic state of randomly chosen regulatory modules (for reasons, see text). (a) Starting from a fully differentiated state, reprogramming factors can perturb any of the remaining 14 positions (for the case of a 4-level hierarchy). Four outcomes are possible depending on the perturbation site: death/arrest, trans-differentiation, de-differentiation or return to the initial cellular state. These outcomes are determined by simulating the system in accord with the rules described in the text and Figs. 2–3. The color code representing genetic and epigenetic states is the same as in Fig. 1 (b) Examples of real trajectories observed in simulations illustrating different temporal evolution of epigenetic and genetic states. Complete cell reprogramming appears as a consequence of several successful de-differentiation events as seen in the second example trajectory. Simulations are performed with parameter values F = 2000; J = 3000; G = 25; H = 40; a = 0; b = 0.3. The color code representing genetic and epigenetic states is the same as in Fig. 1.

Our simulation results suggest a mechanistic explanation for why reprogramming is so rare. When reprogramming factors attempt to change cellular identity by altering the epigenetic state of a previously silenced gene module, the probability of success depends upon the position of this module relative to the one that regulates the terminally differentiated state. We find that the position of the module whose epigenetic state is altered can belong to one of three categories (Fig. 5a).

Suppose this gene module regulates a cellular identity in a different lineage from the terminally differentiated state. In the next interphase, both modules can express proteins as there are no mutually repressive interactions between them. In the subsequent telophase, proteins expressed by each module would favor epigenetic silencing of the other (rule 4). Expression of proteins characteristic of a cell type from a different lineage does not favor reprogramming because it leads to cell death or arrest in our model. Cell death could be mediated by various mechanisms including genetic instabilities if the two open gene modules send conflicting instructions to housekeeping genes. Of course, there is also the chance that the cell will be rescued by stochastic expression of some master-regulatory gene, or that the cell will assume an “intermediate” cell state without master regulation that could be viable, but does not reprogram, such as some arrested states [18]; finally, there is a possibility that two master regulators will not repress each other in full, but some minuscule amount of expression of both will remain thus, arresting the cell. Within the framework of our model we do not distinguish between these possibilities, and classify cells in all these unusual, dead, or arrested states to be dead/arrested.

The gene module whose epigenetic state is altered by reprogramming factors could be in the same lineage as the differentiated cell, but not be its sibling or progenitor. In the following interphase, this module and the one that regulates the terminally differentiated state can both express proteins. In the subsequent telophase, according to our model, protein products of the gene module regulating the terminally differentiated state will favor epigenetic silencing of the module that was turned on by the action of reprogramming factors (rule 4). But, the opposite is not true because the cellular state regulated by the gene module whose epigenetic state was altered by reprogramming factors could potentially differentiate to the terminally differentiated cell type. Thus, the altered gene module will be silenced again, and the cell remains terminally differentiated.

Reprogramming factors could also change the epigenetic state of a previously silenced gene module which regulates an immediate sibling or the progenitor of the terminally differentiated state. In the subsequent interphase, these two gene modules with open chromatin status will not simultaneously express proteins at high levels. This is because gene modules that are “nearest neighbors” in the hierarchy mutually repress each other (rule 2′). If the dominantly expressed gene module (determined stochastically) is the one which regulates a sibling or the progenitor of the terminally differentiated state, then during the next telophase its products will establish epigenetic marks consistent with a new identity (rule 1). Thus, with a probability determined by stochastic effects, a step toward reprogramming can occur via trans-differentiation or de-differentiation.

These arguments suggest that a step toward reprogramming occurs with significant probability only if the epigenetic state of a gene module regulating a sibling or progenitor of the differentiated cell is changed to open chromatin status by reprogramming factors. This is a rare event in our simulations where the set of master regulator genes that determine a cellular identity are considered to be one gene module. In reality, this is even less likely because it requires reprogramming factors to orchestrate changes to a set of master regulator genes synchronously. For successful reprogramming to the ES state, a sequence of such rare events must occur in a particular cell. This is because after a step toward reprogramming occurs, the partially reprogrammed cell is subject to all the constraints discussed above. Therefore, although cellular identity is plastic, reprogramming a terminally differentiated cell to the ES state is rare and requires many cell cycles.

Two examples of how states evolve under the influence of reprogramming factors in our simulations are shown in Fig. 5b. The first example shows a “cell” that does not successfully reprogram, as after a successful trans-differentiation, ultimately the cell is arrested/dead. In the second example reprogramming to the ES state occurs successfully, and it shows an interesting feature. At an intermediate time point, before the ES state is realized, reprogramming factors have turned on expression of the endogenous gene module that regulates the ES state. But this is transient, as this module is quickly silenced. We find that, unless proteins expressed by each gene module can stably repress genes that are distal in the hierarchy of states (rule 4, realized presumably through DNA methylation), expression of endogenous genes that regulate the ES state can occur early and prior to the temporal increase in the number of bivalently marked genes observed during reprogramming. In other words, our model recapitulates the observation that endogenous expression of Oct4 and Sox2 is the last step toward reprogramming only if the “DNA methylation” constraint is long-ranged. Thus, the model suggests that transient blocking of methylation machinery might allow endogenous expression of Oct 4, Sox2, etc., at intermediate time points. This is consistent with the observation that DNA methyltransferase and histone deacetylase (HDAC) inhibitors, such as valproic acid (VPA), an HDAC inhibitor, improve reprogramming efficiency [9].

Our model predicts that reprogramming occurs via a sequence of trans-differentiations to immediate siblings or de-differentiations to immediate progenitors in the hierarchy of cellular states. *Note*, *however*, *that our results do not imply that pure differentiated states will be observed as reprogramming occurs*. Oct4, Sox2, etc., have numerous targets, and so genes from unrelated lineages will transiently be expressed during reprogramming to the ES state (22). But, the entire set of master regulatory genes for a cellular state from a different lineage will not be expressed.

**Figure 6:**
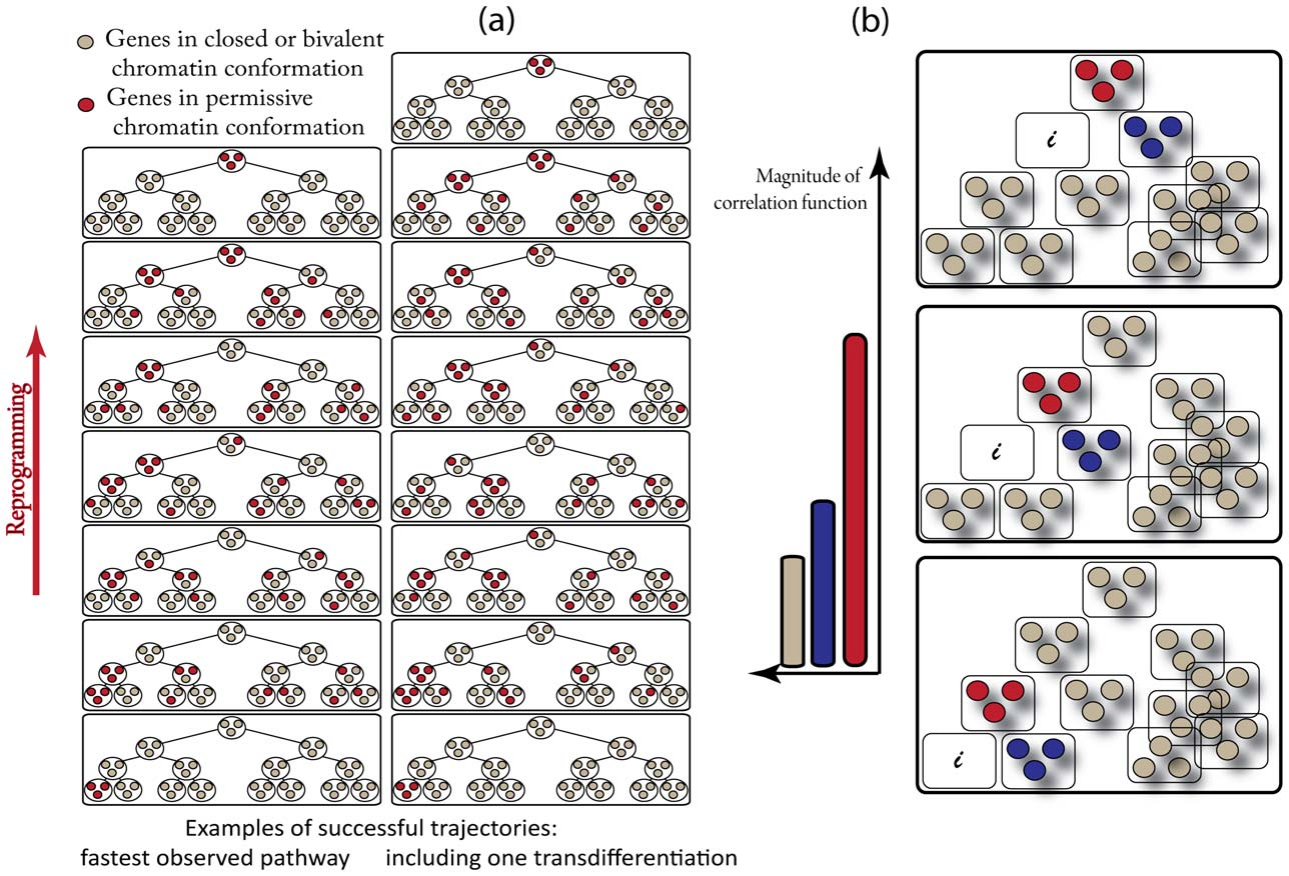
Simulations of a model where each gene module regulating a cellular identity consists of three different genes. (a) In this (similar to the previous) model, individual genes do not interact with each other. Rather modules interact with each other when all of the proteins in a module are expressed. Since reprogramming factors change the epigenetic state of randomly chosen individual genes, several (here: at least three) genes have to be changed to open chromatin status at the same time in order to allow a whole module to be able to express proteins. Examples of simulated trajectories show activation of genes of unrelated lineages during successful reprogramming. Simulations are performed with parameter values F = 2000; J = 3000; G = 25; H = 40; a = 0; b = 0.3. (b) If population averaged expressions of genes during reprogramming can be measured, one can compute a 4-point correlation function (see Eq. 1). This correlation function describes the probability of activation of a given gene after the master regulatory gene module, *i*, was silenced. Then all the genes can be grouped in three groups as our simulation indicates. Thus, the genes defining the most likely paths to reprogramming can be identified as the ones with the highest magnitude of this correlation function. The correlation function was computed by averaging over all successfully reprogrammed trajectories. The colors correspond to the magnitude of the correlation function (as shown on the left).

We illustrate this point by showing computer simulation results from a model where we consider each gene module to be comprised of three individual genes (Fig. 6). Reprogramming factors can attempt to change the epigenetic state of the individual genes randomly as before. However, in this more complex model, if we allow only one gene's epigenetic state to be modified in every telophase, reprogramming becomes so rare that we cannot observe it in a realistic computer simulation time. So, we allowed a larger number of transformations per cycle. Choosing this number to be too large corresponds to overexpression of reprogramming factors, and this severely hinders reprogramming (Text S1, section 2). For the results shown in Fig. 6, we randomly pick 12 genes and change their epigenetic states during each simulated telophase. We assume that the entire set of genes comprising a module must be expressed for its products to regulate the epigenetic or genetic network. This is consistent with combinatorial control of regulation.

Fig. 6a shows two examples of *in silico* cells that successfully reprogram to the ES state. Reprogramming takes place via a sequence of trans-differentiation and de-differentiation events wherein the entire set of genes that regulate a progenitor or sibling of the previous cellular state is expressed. But, the intermediate states are not pure differentiated states as some genes from unrelated lineages are also turned on at the same time (as observed in experiments [18]). If the terminally differentiated state in our simulations is analogous to a B cell, our simulations predict that all successfully reprogrammed cells must transit through an impure state where all the genes regulating the hematopoetic stem cell state are turned on (as in Fig. 6a).

Although beyond the scope of this work, it would be reasonable to test this prediction by applying a cre-lox based lineage-tracing approach. Using one or more stem/progenitor specific promoters that are inactive in the terminal state (e.g., B cell), in combination with a lox-STOP-lox reporter, one could retrospectively determine whether all the resulting iPS cells are labeled and hence have transiently expressed markers of earlier stages within the same lineage. An unrelated cell type, such as fibroblasts, should generate unlabeled iPS cells because it would not be expected to transition through hematopoietic progenitor stages and hence serve as an appropriate control.

The results depicted in Fig. 6 could also potentially be assessed quantitatively in experiments where the temporal evolution of the gene expression patterns of a number of successfully reprogrammed cells is observed. Consider a state where the master regulator genes corresponding to a particular cellular identity are all expressed. One could then ask: when these genes are subsequently silenced during reprogramming, which complete set of master regulatory genes start expressing proteins? One could ask this question at various times during reprogramming and in various successfully reprogrammed cells. This would enable calculation of the following four point correlation function (C):
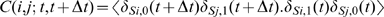
(1)where δ is the Kroenecker delta, t is time, t+Δt is a later instant in time during reprogramming (a cycle in our simulations), i and j are labels of two genes, and S_i_ is either 1 or 0 depending upon whether the i^th^ gene is expressing proteins or turned off.

Our computer simulations predict (Fig. 6b) that, at each stage of reprogramming, the correlation function would have high values for genes from lineages related to the terminally differentiated starting point and low values for genes of unrelated lineages. We hope that this prediction can also be assessed in future experiments. This could involve permanent labeling as mentioned above, or possibly, in the long-term, real-time monitoring of cell state transitions.

## Discussion

To the best of our knowledge, we have developed the first computational model that describes how terminally differentiated cells may be reprogrammed by expression of ectopic genes. This is achieved by a mathematical description of interactions between epigenetic and genetic networks of master-regulatory genes that govern specific cell states. The model also describes differentiation in accord with experiments. Our model describes cellular states as attractors on a generalized landscape of all possible genetic/epigenetic configurations. Cellular states are stable, self-renewing states unless a perturbing signal (either differentiation cue or reprogramming factors are introduced).

As summarized in the table 3, major features of the reprogramming process are explained by our results and the mechanism of reprogramming it suggests. For instance, different cell types can be reprogrammed with the help of the same set of factors [1], [12], [16] because ectopic expression of genes that have many targets (e.g., Oct4 and Sox2) can perturb the epigenetic state regardless of the identity of the starting differentiated cell type. The importance of fast progression through the cell cycle (due to cMyc, Klf4, or p53 knockdown) is because this offers more opportunities for epigenetic transformations during telophase. The important experimental observation that endogenous Oct4 and Nanog expression [2] occurs just prior to complete reprogramming is also recapitulated by our model. The stochastic nature of the reprogramming process [56] and its low yield [2] are because only a few types of trajectories can lead to successful reprogramming, and they are realized rarely by stochastic perturbation of the epigenome by the reprogramming factors. Our model predicts the nature of these rare trajectories to be those that progress through reprogramming via de-differentiation to closely related cell types (immediate progenitors or siblings in the hierarchy). Ways to directly test this prediction are suggested. However, any feature that involves a specific molecular interaction between specific molecules is not described by our model.

In our current model, we consider states with genes that express proteins with conflicting demands to die/arrest. In reality, some of these situations can give rise to steady states that do not arrest or reprogram (such as the recently studied BIV1, MCV8, etc., cell lines) [18]. The ideas emerging from our model are consistent with observations made by manipulating these trapped states.

For example, consider the observation that removing reprogramming factors allows cells from the BIV1 cell line (isolated during reprogramming of B lymphocytes) [18] to reprogram to the ES state. This suggests that overexpression of reprogramming factors prevents these cells from reprogramming to the ES state. Our model suggests that this could be due to two reasons. First, over expression of reprogramming factors (which have many targets) could simultaneously change the epigenetic states of a number of silenced genes to permissive chromatin status. Our simulations of the model shown in Fig. 6 with a large number of such simultaneous transformations (e.g., 22 at a time, rather than 12 at a time used for Fig. 5) prevents successful reprogramming because of the large probability of obtaining dead or arrested states. As noted above, one of these states that cannot reprogram could correspond to the BIV1 cells.

Secondly, our model describes how lowering expression of reprogramming factors in BIV1 cells could enable reprogramming. In our simulations, we consider proteins expressed during each interphase to act on the epigenome to reach a new balance which then leads to a corresponding protein expression pattern before another epigenetic transformation can occur due to the action of reprogramming factors. This is analogous to assuming that the reprogramming factors can act to change the epigenetic state of a set of master regulator genes rarely. If reprogramming factors are grossly overexpressed, this would not be true. So, before a new protein expression pattern could be expressed consistent with a newly acquired epigenome (say, de-differentiation to a progenitor), another epigenetic transformation would occur, and the whole cycle would start again. Simulation results showing this effect upon overexpression of reprogramming factors are depicted in Fig. 3B in Text S1. Removing reprogramming factors could potentially allow reprogramming of cells trapped in such an infinite loop.

Our low-resolution model for the architecture of genetic and epigenetic regulatory networks that determine how cellular identities change is consistent with diverse observations (Table 3). In formulating this model, we ruled out many models that were inconsistent with known experimental results, but we cannot rule out all other possible models. Therefore, the predictions of the model (noted earlier) need to be experimentally tested (perhaps in ways that we have suggested) to either falsify it or encourage studying it further. If tested positively, the suggestions emerging from our model regarding ways to enhance reprogramming yields should be further explored. It would also be interesting to study other transcription factor induced cell state conversions [57], [58] within the conceptual and computational framework we have developed for how cellular identity is transformed. In particular, recent results of direct conversion between exocrine and endocrine cells through ectopic expression of three alternative transcription factors [59] should be examined.

It would be interesting to further investigate several assumptions adopted in the model for the lack of specific information about individual master-regulatory modules. For example, maximum expression levels of different master-proteins within different modules could differ, as well as coupling between genetic and epigenetic networks could be different for different modules. Also, we assumed that every simulated cell (as represented by a simulated trajectory) has the same level of expression of reprogramming factors while in reality cells can be transfected in a heterogeneous fashion. Also, the difference in viral integration sites in different cells could lead to the different expression levels of exogeneous genes thus making effect of reprogramming factors heterogeneous across the population. In a sense then, we have studied those cells which have expressed reprogramming factors at levels above a threshold. It would be interesting to further explore the consequences of such heterogeneity. Another avenue for further exploration lies in defining the notion of time during the reprogramming process, in this work cell cycling has been adopted as a measure of time required for reprogramming while in reality cells cycle with non-equal rates determined from some form of cell division rate distribution (simplest form would be an exponential distribution). It would be interesting to see applicability of the 4-point correlation function based analysis for the situation when cell cycling rates are not identical. Finally, de-silencing action of reprogramming factors is assumed to be distributed randomly. It would be interesting to consider situations when de-silencing distribution is not uniform across the hierarchy. It is possible that non-uniform distributions can improve the reprogramming efficiency.

From the standpoint of statistical physics, our model couples a Potts model with short and long-ranged interactions in external fields (Eq. 2) with an Ising model with short-ranged interactions in an external field (Eq. 3). It may be fruitful to develop a deeper field-theoretic understanding of such models.

## Methods

All simulations are carried out with the help of two hierarchical lattices because two lattices are required to properly describe the cell state as shown in Fig. 1b. In the simulation code provided in Text S2, we consider 4 levels in the hierarchy (such as the one in Fig. 1b). Other possibilities (3 and 5 levels) have been considered also.

The epigenetic lattice has a discrete epigenetic state associated with each node (−1,0,+1). S^epigen^ = −1 corresponds to closed chromatin, S^epigen^ = 0 corresponds to bivalent chromatin and S^epigen^ = +1 corresponds to open chromatin. Genetic lattice describes expression of proteins from master-regulatory modules. It has discrete gene expression states associated with each node (0, +1). S^gen^ = 0 corresponds to the absence of any protein expression from the given gene, S^gen^ = +1 corresponds to the maximum protein expression from the gene. In the course of simulation, cell states change in response to random epigenetic perturbations according to the rules described above (see Table 1 for summary). There are two possible endpoints for the simulation procedure: either the cell will assume a dead/arrested state, in which case the simulation stops; or it will, as a consequence of a random sequence of epigenetic transformations, be reprogrammed to the ES-state, which is indicated by the stable turning on proteins expressed by the of ES-regulatory module. In the latter case we stop the simulation procedure manually because, according to experimental observations [2], stable expression of endogenous Oct4 suppresses expression from the exogenous locus, thus preventing future action of reprogramming factors.

**Figure 7:**
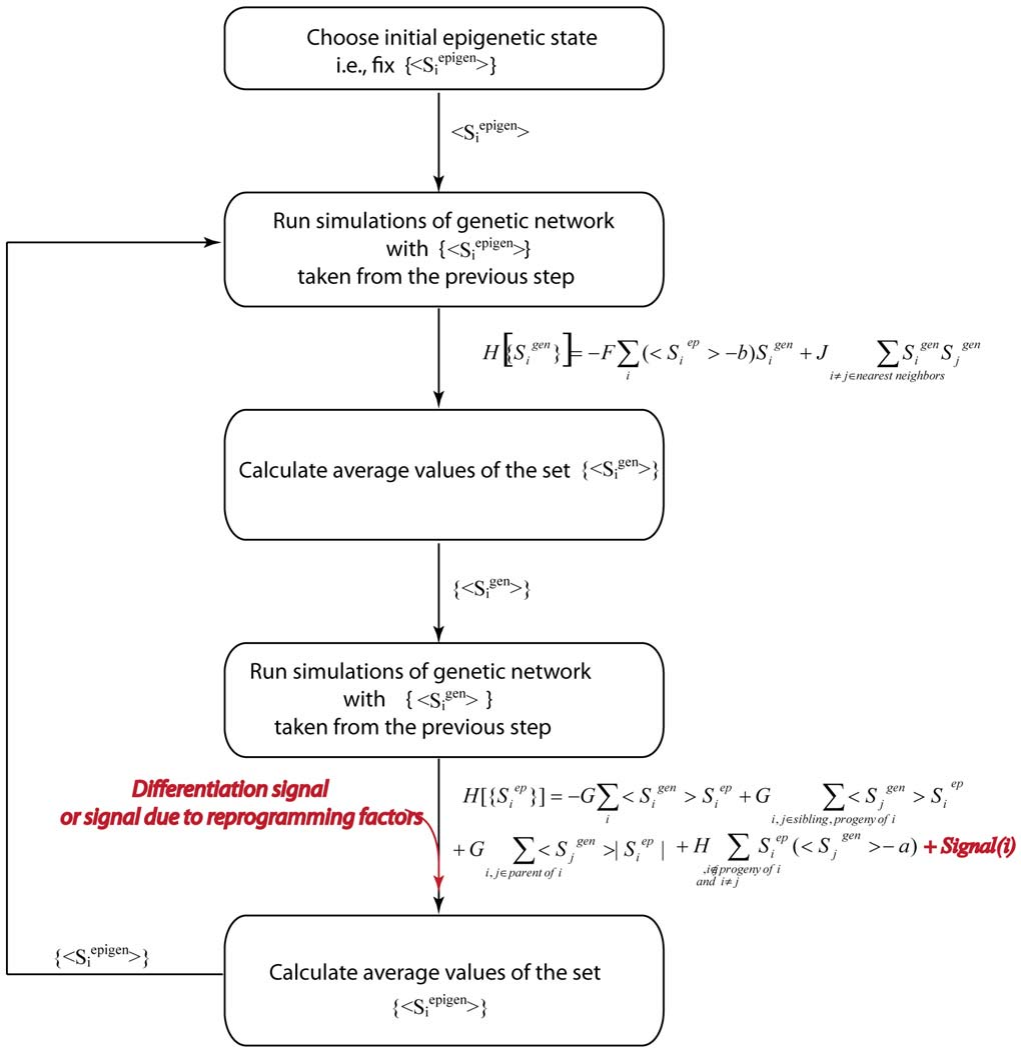
Flow chart of the simulation procedure. The simulation essentially mimics progression through the cell cycle in accord with Fig. 2. In each phase of the cell cycle, interactions within and between genetic and epigenetic lattices are enforced through the Hamiltonians of Eq. 2 and 3. Mathematical structure and choice of parameters are such that rules depicted in Fig. 3 are obeyed. For analysis of sensitivity to parameter variations see Text S1.

In order to initialize simulations one has to specify either the epigenetic or genetic state of the lattice (see Fig. 7). If we start by specifying the protein expression pattern, computer simulations are carried out to determine the epigenetic state that is realized in telophase. A Monte-Carlo simulation algorithm is used in accord with the following Hamiltonian, with its four terms representing rules 1–4 (see Model development), respectively:
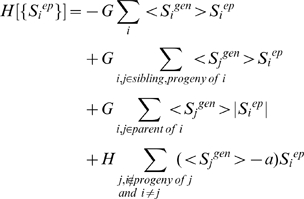
(2)S_i_^ep^ denotes the epigenetic spin state of the i^th^ module, and S_i_^gen^ specifies the protein expression level of the i^th^ module. The angular brackets denote the average expression level of the j^th^ module obtained during the preceding interphase, and could include protein products of ectopic genes or signaling events. |S_i_^ep^| represents the absolute value of S_i_^ep^. The quantity G is a positive parameter that represents the strength with which the protein atmosphere can modify the epigenetic state by altering histone marks. H is a positive parameter that represents the strength of the DNA methylation constraint. The quantity, a, is a positive constant that favors values of S_i_^ep^<a if proteins expressed by gene, j, are present. As detailed in the Text S1 (see section 2), the results of our simulations are inconsistent with experimental results if H is not greater than G. As long as H>G, our qualitative results do not depend upon the specific values of these parameters. The specific value of *a* does not affect qualitative results. Results presented in the main text are for a = 0, and G = 25, H = 40 (in units described below).

During simulation of the telophase, the epigenetic state S^epigen^ of each module fluctuates. The output of the telophase simulation is <S^epigen^>, an average of these fluctuating values for each node of the lattice (i.e. for each module). Because we have a discrete representation for the epigenetic marks (+1, 0, or −1) while actually each gene bears multiple marks, using the average allows us to reflect intermediate levels of positive and negative histone marks on a gene. For example, an average value near zero for the epigenetic state of a gene module implies that both positive and negative marks are present on histones associated with it, a value close to one represents an open chromatin state, *etc*.

Average values of epigenetic state serve as input for simulation of interphase. If <S^epigen^>∼1 (gene is epigenetically available), than it will favor protein expression during the interphase in accord with the rules depicted on Fig. 3a. Similarly, if two neighboring states are epigenetically available, only one protein will be expressed due to mutual repression of neighboring master-regulators. Separate Monte Carlo simulations are carried out to establish gene expression patterns during interphase. The following Hamiltonian, with the two terms in it corresponding to rules 1′ and 2′ (see Model development), respectively, is used:
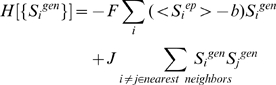
(3)The angular brackets denote the average value of epigenetic state of the i^th^ module obtained during the preceding telophase. F is a positive constant that represents how strongly a protein is expressed or repressed if it is in open chromatin state or in heterochromatin, respectively. The parameter, b, is a positive constant; protein expression is favored if <S_i_^ep^>>b. Note that the form of the first term in Eq. 3 implies that protein expression is more strongly repressed if a gene is packaged in heterochromatin compared to if it is bivalently marked. J represents the strength of mutual repression by other proteins. As detailed in the Text S1 (section 2), our results are inconsistent with experiments if J is not greater than F. As long as J>F, the specific values do not affect qualitative results. As long as the parameter b is larger than the typical size of fluctuations in <S_i_^ep^> (∼0.1), the specific value of b does not affect qualitative results. Results presented in the main text correspond to b = 0.3, and F = 2000, J = 3000 (for units, see below).

Values of S_i_^gen^ fluctuate during this Monte-Carlo procedure. The output of the simulation of the interphase is <S_i_^gen^>, which represents the average expression level of the regulatory protein in the interphase. These averages are further used in the next telophase simulation, thus, completing the cycle.

The Monte-Carlo algorithm is standard [60]: the lattice spins (+1/0/−1 on epigenetic lattice; +1/0 on genetic lattice) are initialized randomly. The move consists of 1) randomly choosing the node on the lattice; 2) randomly deciding on the choice of new value of S_i_ for this node (i.e. if S_i_^epigen^ was 0 then it can become −1 or +1 with equal probability); 3) energy for this configuration is computed according to the appropriate Hamiltonian; 4) attempted changes in state are accepted with probability equal to min [1, exp {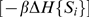]. The parameter,β, is analogous to inverse temperature used in simulation of thermal systems, and sets the scale for the parameters, F, G, H and J. If we pick this effective temperature to be too high (β≪F, G, H, J), the system is disordered; specific cellular identities are not established and the model has no biological significance. We use β = 1 for results reported in the main text. During each phase, the Monte-Carlo procedure is carried out until running average values of <S_i_^ep/gen^> stop changing along the trajectory; *i.e.*, they converge to a single well-defined value. For the reported parameters (Table 2), 50,000 updates are sufficient for accurate averaging during each phase.

A computer code written using the C++ language is provided as Text S2 allows calculation of all the results we report. For details regarding the output and input formats see the Text S1.

## Supporting Information

Text S1 Supporting material including parameter sensitivity analysis and supplementary figures
(1.34 MB DOC)

Text S2 C++ source code for simulations of reprogramming trajectories
(0.01 MB TXT)
