# Supplementary material for: A Model for Genetic and Epigenetic Regulatory Networks Identifies Rare Pathways for Transcription Factor Induced Pluripotency
Source: PLoS Comput Biol. 2010 May 13;6(5):e1000785. doi: 10.1371/journal.pcbi.1000785 (PMC2869311; doi:10.1371/journal.pcbi.1000785)
Supplement: Text S1 — Supporting material including parameter sensitivity analysis and supplementary figures (1.34 MB DOC) [file pcbi.1000785.s001.doc]

Supplementary information for

**A model for genetic and epigenetic regulatory networks identifies rare pathways for transcription factor induced pluripotency**

Maxim N. Artyomov^1^, Alexander Meissner^2^, and Arup K. Chakraborty^1,3-5,*^

Departments of Chemistry^1^, Chemical Engineering^3^, and Biological Engineering^4^

Massachusetts Institute of Technology, Cambridge, MA 02139.

Ragon Institute of MGH, MIT, and Harvard^5^.

Department of Stem Cell and Regenerative Biology, Harvard University and Harvard Stem Cell Institute, Cambridge, MA 02138; Broad Institute of MIT and Harvard, Cambridge, MA 02142.^2^

*address correspondence to [arupc@mit.edu](mailto:arupc@mit.edu)

**1. Parameter sensitivity studies**

Here we discuss how changes in the six parameters in our model (Eqs. 2 and 3 in the main text) affect the results of our computer simulations. The results shown in the main text correspond to the following parameter values: F=2000; J=3000; G=25; H=40; *a*=0; *b*=0.3

The effective temperature used in the simulations is T=1. For any other choice of the temperature, T^new^, the parameters F,J,G,H have to be linearly scaled; i.e., F^new^=F*T^new^ , *etc*, for the simulation results to be invariant.

The simulations results reported in the main text do not change qualitatively as long as the parameters lie in the following ranges: H>G>>T & J>F>>T, *b* lies between 0.1 and 0.5, and *a* between 0 and 0.6. Below, we provide a detailed description of why these parameter ranges are appropriate.

*1a. Parameters involved in simulation of the epigenetic network (Eq. 2): H, G, and a.*

The parameters, G and H, describe the coupling between genetic and epigenetic networks. For example, G is the strength with which a value of <S_i_^gen^> = 1 (i.e., high protein expression by the i^th^ gene module) favors a value of + 1 for <S_i_^epigen^> (i.e., open chromatin) during simulation of the epigenetic network. H is the strength of the methylation constraint. It determines the strength with which proteins expressed by a gene module favor methylation of another gene module according to rule 4 in the main text. We have carried out simulations with H>G, H~G, and H<G. The simulation results reported in the main text correspond to H>G. The other two parameter regimes lead to results that are inconsistent with experimental findings as described below. Consider situations where G and H are approximately of the same magnitude, H ~ G. Now, when proteins expressed by two distal gene modules are expressed simultaneously (for example, as a result of reprogramming factors’ action), this leads to a tug-of-war which is resolved in several (typically, 2-3) cell cycles. Because H~G, stochastically, only one of the modules will be silenced when cell will achieve epigenetically/genetically balanced state. When H=G and two proteins are expressed at exactly the same level, the methylation (H) and self-support (G) will exactly cancel each other and in the next cycle two protein will be expressed at exactly the same levels as in the previous cycle. However, due fluctuations, proteins never have *exactly* same expression levels. And the slightest differences in protein levels is amplified in the next cycle because H-G is positive for one protein (hence, this protein is more suppressed) and negative for the other protein (hence, it will be epigenetically more available in the next cycle).Thus, in several cycles balanced epigenetic/genetic state with only one master-protein expressed is achieved. (In the simulations reported in fig.S1a we perform epigenetic perturbation every 4 cycles in order to allow for cell state equilibration time). If we allow epigenetic perturbations to be more frequent, in this circumstance, each expressing gene module experiences methylation constraint from multiple other modules which results in silencing of all genes (death/arrest).

Therefore, in this parameter regime, random lineage switches occur during reprogramming and no specific path for reprogramming trajectories can be identified (see Fig.S1a). For example, lineage switches can occur from a fully differentiated state to a state in a different lineage that is almost fully reprogrammed. This corresponds to sudden changes in epigenetic patterns of the cell, rather than a continuous evolution of the number of bivalent domains. For G=H, we find that such cross-lineage jumps are dominant in our simulations, occurring 21 times more frequently than gradual evolution of epigenetic patterns in successfully reprogrammed trajectories. Thus, when H~G, “shortcuts” are dominant during simulations of reprogramming, leading to large and rapid “jumps” from a differentiated state to a nearly reprogrammed state. This is inconsistent with experimental observation of timelines of reprogramming with distinct changes in cell appearance and cell markers (1). Note that sudden jumps of the sort we see in our simulations with G~H have been observed upon treating fibroblasts with a drug called AZA (2), which causes global demethylation (i.e., removes the methylation constraint). These large jumps do not lead to reprogramming. Based on these considerations, we believe that our simulation results for G~H are not consistent with experimental observations.

If H<G, because the methylation constraints are weak, we often see stable expression of the endogenous gene module regulating the ES state after one or two cycles, and without transitioning through intermediate states. For example, even if H is only 3 times smaller than G, 8% of trajectories exhibit stable induction of the gene module regulating the ES state after the very first cycle of epigenetic perturbations. This is inconsistent with experimental results showing that endogenous expression of Oct4, Sox2, etc., never appears before at least day twelve, and that reprogramming is associated with a gradual evolution of cell appearance and markers (1). For these reasons, we focused on the parameter regime H>G, for which the results reported in the main text are qualitatively robust. For example, if H = 60 and G = 35, rather than H = 40 and G = 25 as in the main text’s figures, the reprogramming efficiency for a 4-level hierarchy is ~5 out of 10^4^ cells and the qualitative pathways followed by successfully reprogrammed cells remains the same as discussed in the main text.

The parameter, a, describes the minimal level of gene expression that allows a particular gene module to exert methylation constraints on other genes. In our simulations, *a*=0, which means that, as long as protein is expressed, it can methylate other gene modules with a strength related to its expression level. Changing the parameter *a* between 0 and 0.6 does not alter qualitative results. For example, if a = 0.5, rather than 0 as in the main text, the reprogramming efficiency for a 4-level hierarchy of states is ~6 out of 10^4^ cells, and the qualitative pathways followed by successfully reprogrammed cells remain the same as discussed in the main text.

*1b. Parameters involved in the simulation of the genetic network: F, J and b.*

Parameters F and J describe the coupling between the epigenetic and genetic network. Parameter F is the strength with which open chromatin (positive <S_i_^epigen^>) favors gene expression (positive S_i_^gen^) during the simulation of the genetic network. Parameter J describes the strength with which proteins expressed by two neighboring gene modules in open epigenetic states mutually repress each other. The choice of F and J is dictated by our assumption that two neighboring genes with open chromatin should with high probability express only one out of two proteins. This is to be consistent with experimental evidence that genes modules responsible for the competing lineages are mutually repressive (3). For this to be true, the parameter J needs to be larger than F.

Stochastically, during simulation of the genetic network, transitions can occur from a state where one of two mutually repressive gene modules is expressed to one where the other is expressed. F and J must be much larger than the effective temperature of the simulations (T) in order to minimize such transitions during the simulation time as this would result in both proteins being simultaneously expressed. If the two gene modules thus expressing proteins are immediate siblings in the hierarchy of states, then rules 3 and 4 in the main text would result in silencing of both gene modules, leading to cell death/arrest. If the two gene modules were progenitor and immediate progeny, then the cell would remain in a terminally differentiated state because of the asymmetry of the methylation constraint for states in the same lineage. Fig S3 shows an example of the latter situation for simulations carried out with values of F and J that are too small. Thus, if F and J are not sufficiently large, reprogramming is not possible. For intermediate values of F and J we find that reprogramming probability decreases (for F=1000 and J=2000 only about 4 cells out of 10^5^ cells reprogram successfully), but the qualitative nature of the temporal pathways followed by successfully reprogrammed cells remain the same.

Protein expression by gene module *i* is favored when the value of <S_i_^epigen^> representing the chromatin state of this gene is greater then *b*. The value of *b* has to be more than zero to ensure stability of the stem cell state in the absence of self-induced differentiation (e.g., when LIF, etc., are present in the medium). Typical simulations show that, in the ES state, <S^epigen^(ES gene module)> ~1 and <S^epigen^(all other gene modules)> fluctuates between -0.1 and +0.1. The parameter, b, must be larger than the size of these typical fluctuations in order to prevent spontaneous differentiation. Our qualitative results do not change if b lies between 0.1 and 0.5. For example, if b = 0.4, rather than 0.3 as in the main text, the reprogramming efficiency for a 4-level hierarchy of states is ~6 out of 10^4^ cells, and the qualitative pathways followed by successfully reprogrammed cells remain the same as discussed in the main text.

**2. Effect of increasing the frequency of action by reprogramming factors**

Fig. S3a details the events that occur during one-level de-differentiation in our simulations with parameters that are consistent with experimental observations (see above). In our simulations, it takes two cell cycles to complete a successful de-differentiation or trans-differentiation. During the first cycle, reprogramming factors cause a new perturbation to the epigenome in the telophase and the perturbed epigenome controls protein expression in the subsequent interphase. The resulting protein expression profile may not be in balance with underlying epigenetic state. During the subsequent telophase (which begins the second cycle), a new balance between the epigenetic and genetic states of the cell is established. Hence, in the subsequent interphase, the protein expression reaches accord with the prevailing epigenetic state. Note that in experiments, it is likely that more time (cell cycles) is required in order to balance the genetic and epigenetic states. For example, in nuclear transfer experiments it takes 12 to 20 cycles to achieve global demethylation of somatic DNA (1, 4).

If the reprogramming factors are overexpressed, they will have a higher probability of altering the epigenetic states of gene modules more frequently than once in two cell cycles. In this situation, our simulations predict that reprogramming is not possible because cells will never be able to balance their genetic and epigenetic networks, as is shown on Fig.S3b. Any endogenous expression of Oct-4 or Nanog will only be observed transiently, because further epigenetic perturbations occur before the ES state can be stably established.

Overexpression of reprogramming factors should also lead to an increase in the number of genes that can be epigenetically altered in every cell cycle. In our simulations, this means that a greater number of genes would be epigenetically modified during each perturbation of the epigenome. The effects of this can be evaluated in a model where each module consists of three individual genes (Fig. 6 in main text). If only 12 genes are altered in a cycle (as in main text), 15 out of 100, 000 “cells” reprogram. But if 15, 20, or 22 genes are altered per cycle, out of 100, 000 “cells”, only 5, 2, and 0 cells reprogram. Thus, overexpression of reprogramming factors hinders reprogramming.

**3. C++ code that illustrates reprogramming process**

C++ code is provided as supplementary file “reprog.cpp” (Text S2). The code allows one observe reprogramming trajectories. (Simply compile and then run the code after compiling).

Simulation shows progression of the cell states starting from terminally differentiated state under the action of reprogramming factors (they are turned on in the second cell cycle after stability of the terminal state by itself is illustrated).

The simulation outputs the average values of the genetic and epigenetic variable at each cycle in accord with the simulation flow chart.

So, for fully differentiated state genetic and epigenetic networks will look like:

GENETIC

0

0 0

0 0 0 0

1 0 0 0 0 0 0 0

EPIGENETIC

-1

-1 -1

-1 -1 -1 -1

+1 -1 -1 -1 -1 -1 -1 -1

**Terminally differentiated state**

The GENETIC diagram indicates that only proteins of “bottom left corner” master-regulatory gene module are expressed. And EPIGENETIC diagram indicates that chromatin open only for “bottom left corner” master-gene, while the rest of master genes are in the closed chromatin state.

Note that this state is stable state of the cell, “attractor” in the space of the cellular states, because it will renew itself, provided that cell is forced to proliferate.

Fully differentiated state will be recognized in the program output as having the following genetic/epigenetic structure:

GENETIC

1

0 0

0 0 0 0

0 0 0 0 0 0 0 0

EPIGENETIC

+1

0 0

0 0 0 0

0 0 0 0 0 0 0 0

**ES state**

Simulation begins in the fully differentiated state of the 4 levels hierarchy of cell states.

Beginning from the second cell cycle reprogramming factors start acting. They randomly perturb epigenetic structure of the terminally differentiated cell. In the output one can see that randomly chosen epigenetic module will have its “-1” state flipped to “+1” state. Then, during the next cell cycle epigenetic and genetic will find assume balanced state, which in the majority of cases results in the completely silenced, unviable state such as follows:

GENETIC

0

0 0

0 0 0 0

0 0 0 0 0 0 0 0

**Dead/arrested**

EPIGENETIC

-1

-1 -1

-1 -1 -1 -1

-1 -1 -1 -1 -1 -1 -1 -1

This is indicative of cell arrest/cell death situation.

In order to start new reprogramming trajectory, one can simply restart the compiled code.

In the few cases one will observe one-level de-differentiation and in about 5-10 out of 10,000 cases one will see the complete reprogramming from terminal fully differentiated state to the ES cell state.

**4. Population behavior of the cells**

On Fig.S5 the diagram depicting transitions between stable cell populations under the action of reprogramming factors is presented. The starting point is terminally differentiated state depicted on the bottom of the picture. Possible transitions from this state are indicated by arrows. Probability to experience a particular transition is indicated by the % value on top of the corresponding arrow. For example, starting from the population of terminally differentiated cells, only ~7 % of cells successfully de-differentiate one level, while 67 % of cells either die or get arrested in the intermediate state. Further, one sees that out of one level de-differentiated cells only ~7% dedifferentiate one level further etc, thus giving very low total yield of reprogrammed cells at about 0.04%.

**References**

1. Jaenisch, R., and R. Young. 2008. Stem cells, the molecular circuitry of pluripotency and nuclear reprogramming. *Cell* 132:567-582.

2. Meissner, A., T. S. Mikkelsen, H. C. Gu, M. Wernig, J. Hanna, A. Sivachenko, X. L. Zhang, B. E. Bernstein, C. Nusbaum, D. B. Jaffe, A. Gnirke, R. Jaenisch, and E. S. Lander. 2008. Genome-scale DNA methylation maps of pluripotent and differentiated cells. *Nature* 454:766-U791.

3. Cinquin, O., and J. Demongeot. 2005. High-dimensional switches and the modelling of cellular differentiation. *Journal of Theoretical Biology* 233:391-411.

4. Simonsson, S., and J. Gurdon. 2004. DNA demethylation is necessary for the epigenetic reprogramming of somatic cell nuclei. *Nature Cell Biology* 6:984-990.

**Table S1**. Summary of the efficiency of reprogramming in different parameter regimes.

|  | **G<H** | **G~H** | **G>H** |
| --- | --- | --- | --- |
| Efficiency of reprogramming  (out of 10^4^ cells) | 4 | 84 | 0 |

**Table S2**. Dependence of reprogramming efficiency on number of genes perturbed during action of reprogramming factors for the model where each master-regulatory module consists of 3 genes.

| Number of genes perturbed during action of reprogramming factors | **8** | **10** | **12** | **15** | **20** | **22** |
| --- | --- | --- | --- | --- | --- | --- |
| Efficiency of reprogramming  (out of 10^5^ cells) | 6 | 10 | 15 | 5 | 2 | 0 |


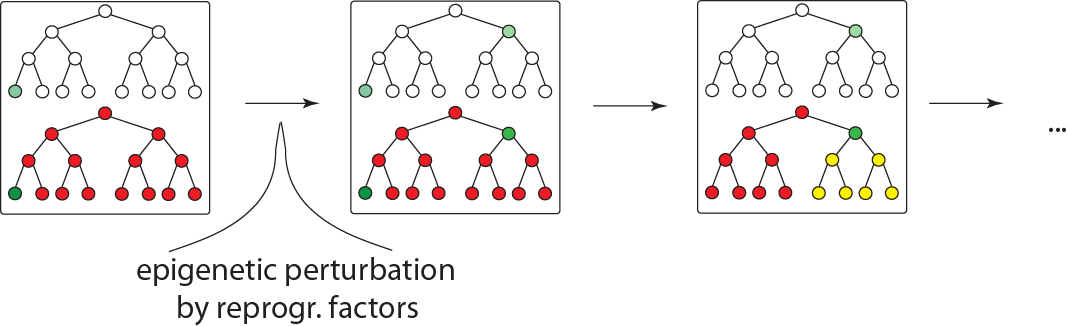


**Fig. S1a**: Example of a part of a simulation trajectory for H=G illustrating large and fast cross-lineage jumps from a terminally differentiated to an almost reprogrammed state. As noted in section 2.a, such jumps are dominant when H~G. (This simulation was carried out with F=2000; J=3000; G=25; H=25; *a*=0; *b*=0.3). The color code representing genetic and epigenetic states is the same as in Fig.1 of the main text.


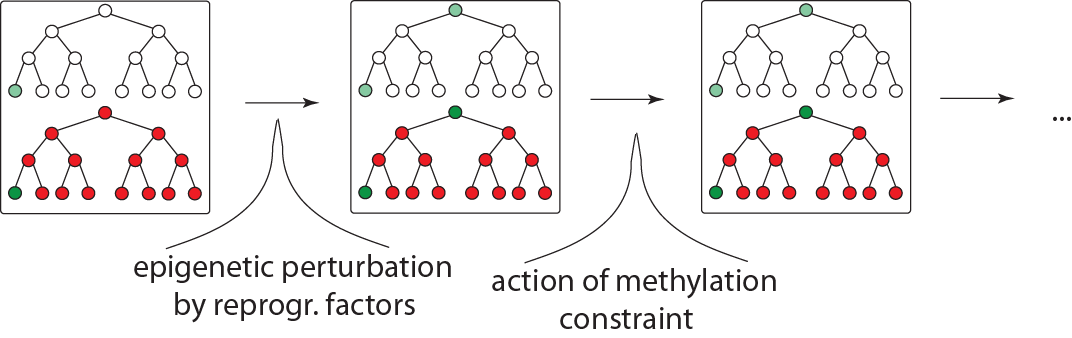


**Fig. S1b**: Example of a part of a simulation trajectory for G>H (in this case G>3H). The gene module regulating the ES state is turned on stably during the first epigenetic perturbation. This simulation was carried out with F=2000; J=3000; G=25; H=8; *a*=0; *b*=0.3. The color code representing genetic and epigenetic states is the same as in Fig.1 of the main text.


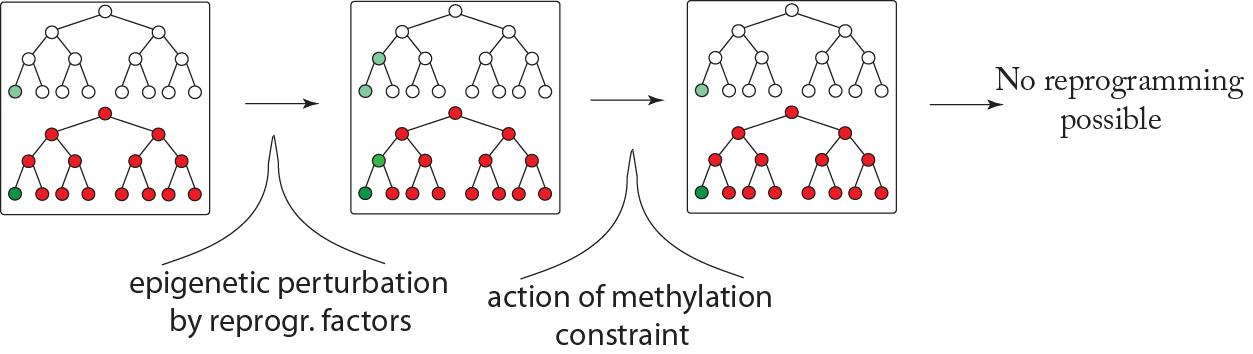


**Fig. S2**: Example of a part of a simulation trajectory with small values of F and J. The simulation was carried out with F=200, J=300, G=25; H=40; *a*=0; *b*=0.3. The color code representing genetic and epigenetic states is the same as in Fig.1 of the main text.


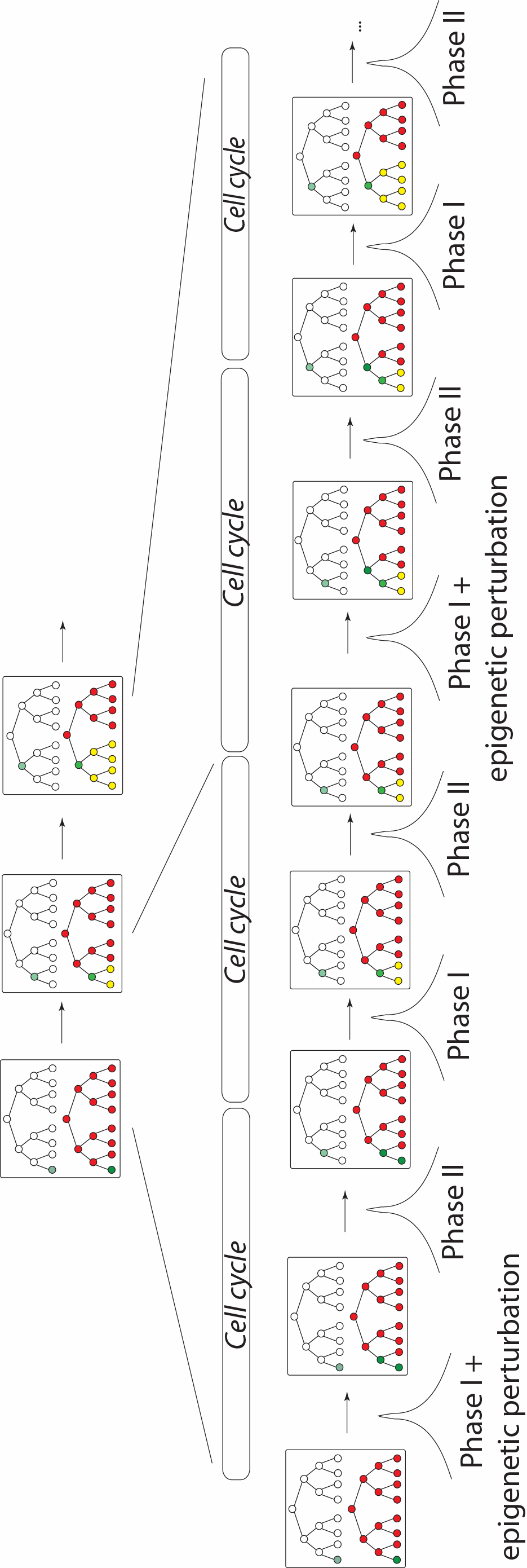


**Fig. S3a:** Example of a typical trajectory obtained from our simulations showing that it takes several cell cycles (in our simulations, 2 cycles) to achieve one level of reprogramming in a stable manner. This simulation was performed with parameter values: F=2000; J=3000; G=25; H=40; *a*=0; *b*=0.3. The color code representing genetic and epigenetic states is the same as in Fig.1 of the main text.


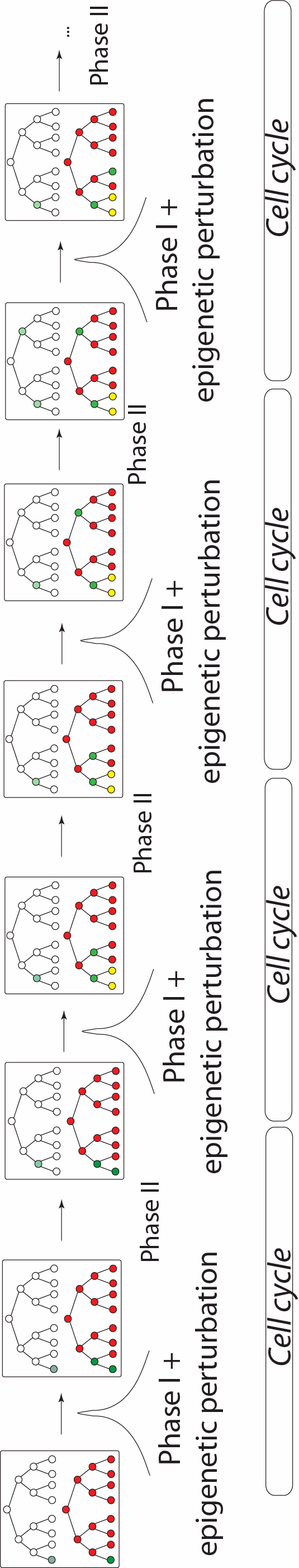


**Fig. S3b**: Example of a typical trajectory from our simulations where, because reprogramming factors are overexpressed, they can act at every cycle, as opposed to the every two cycles (as in panel (a)). This renders reprogramming impossible for reasons described in the main text. This simulation was performed with parameter values: F=2000; J=3000; G=25; H=40; *a*=0; *b*=0.3.


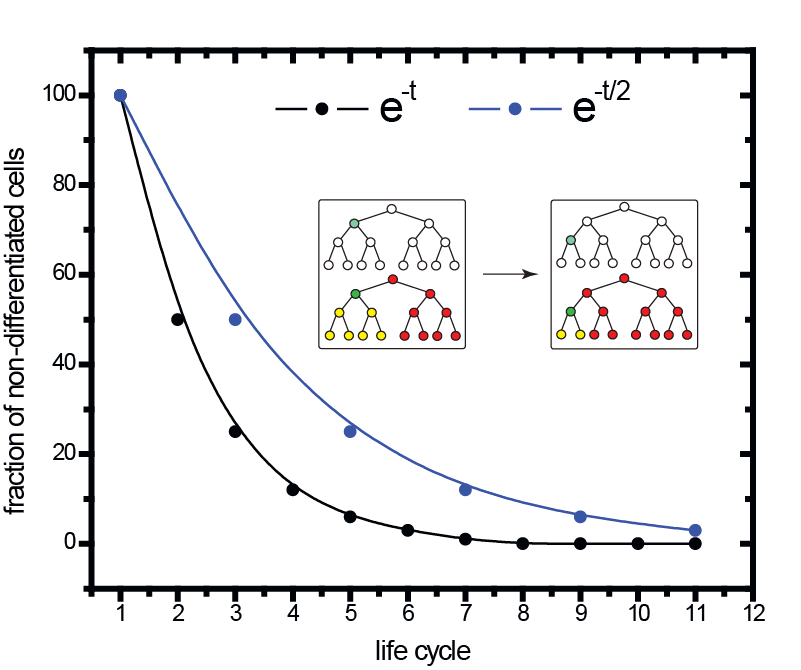


**Fig.S4:**Dynamics of cell differentiation upon receiving cues of different strength. Our simulations show that the progenitor cells differentiate in accord with first order kinetics, with the lifetime of progenitor cells depending on the signal strength. The blue curve describes the behavior of a cell population which received a signal that is twice as weak as the population represents by the black line. Simulations are performed with parameter values F=2000; J=3000; G=25; H=40; a=0; b=0.3.


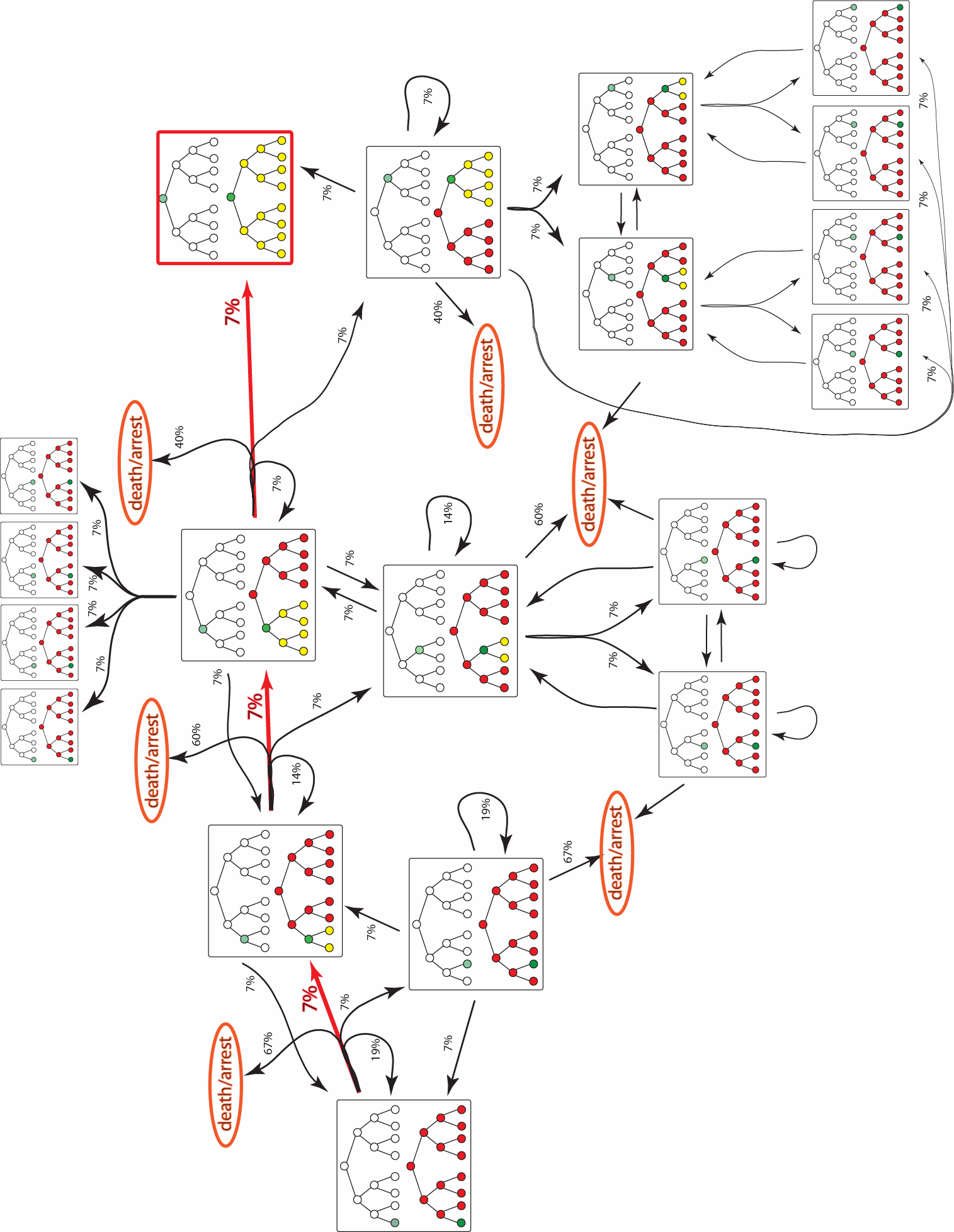


**Fig.S5:** The diagram depicting transitions between stable cell populations under the action of reprogramming factors. The starting point is terminally differentiated state depicted on the bottom of the picture. Possible transitions from this state are indicated by arrows. Probability to experience a particular transition is indicated by the % value on top of the corresponding arrow.
